# What Is Dentistry—The Dignity of the Profession—The Standard to Which It Should Be Raised

**Published:** 1877-10

**Authors:** J. D. Miles


### WHAT IS DENTISTRY—THE DIGNITY OF THE PROFESSION—THE STANDARD TO WHICH IT SHOULD BE RAISED.

BY DR. J. D. MILES.

*Read before the Mississippi Dental Society.*

*To the President and Fellows of the Mississippi State Dental Association.—*

GENTLEMAN: I had hoped till the last moment, to be able to participate with you in the benefits of the third Anniversary of our Association, but I regret to say, that unavoidable circumstances deny me the coveted privilege. My desire to contribute something to vary, if not to add interest to the exercises of the meeting, is my apology for offering a few, hasty, desultory thoughts on the ever fruitful subject of dentistry, a subject too little studied and too little understood by the general student.

What, let me ask, without pausing for an answer, does the profession understand by the term dentistry? Has it to-day the same signification it had in the days of Herodotus, who was the first historian to note the fact, that the Egyptians had a class of men whose special office was to take care of the teeth? He unfortunately does not give a detailed description of them, and has thus left us in the dark as to the perfection this art preservative had attained in his day, but from the fact that the Egyptians had practiced dentistry from remote ages, it is reasonable to infer, they were skilled and proficient in their profession.

It is interesting through the medium of history, to interview the old philosophers, and ask them what they knew about teeth and their preservation, and to trace the successive steps of the progress of Dental Science from Hypocrates, the father of Medical Science, to Aristotle, Galen, Ambrose, Pare, Malpighi, Winslow, John Hunter, and other celebrities of our own day, running through a period from the present back 350 years before Christ. Well may the student feel his heart glow with professional pride, as from his researches he finds how infinitely dentistry to-day, in all its appointments, surpasses all ages of the past, and well may he be excused, if from the inspiration of the moment, he concludes that dentistry has to-day reached the acme of perfection. But this is an error that older and wiser heads must labor to eradicate, for great as have been the achievements even within our own experience, much more must be accomplished before this beautiful science reaches perfection. There is something pleasant in the fact, that in no country has dentistry made so rapid strides towards perfection as in the United States. It is here, also, that dentistry offers her richest rewards to students of natural capability, talent and energy. And let no one enter this field of labor with the hope of reward, who does not possess these requisite qualifications, otherwise a blot will be cast on the fair escutcheon of our profession, and our ears will be made to tingle with jeers and opprobrium heaped upon dentistry. It is a fact much to be regretted, that nothing has so retarded the upward progress of dentistry towards professional equality with the medical profession, as incompetent practitioners, or quacks, commonly known and designated as tooth carpenters.

Professional pride, gentlemen, calls loudly on us to eradicate this evil, an evil fraught with mischief alike to the profession and the community at large, as the ever credulous multitude have more faith in the oily-tongued charlatan than in the meritorious artist, too modest to blow his own trumpet. But the question arises, how shall we rid ourselves of these dead-weights, that prevent our rising to as elevated and honorable a position, professionally, as we should? The work is herculean; to accomplish it, we must labor to make our Association a power in the land, that shall be felt and respected. We must present this subject to the thoughtful attention of our legislators, and urge them to enact a law prohibiting all incompetent persons, or quacks, from practicing dentistry within the limits of this State; and then we must see to it, that the law shall be faithfully executed; and further, we must try to freeze them out of the practice, so to speak, by invariably giving them the cold shoulder, and having no professional intercourse with them whatever. Should this line of policy be strictly carried out, our profession will be rid of this incubus, and will rapidly raise to honorable recognition by the medical profession. But our mission will not be filled, when we shall have obtained from the legislature all we ask of it; we must go a step further, and labor to induce the general government to have a proper appreciation of the importance of our profession, and to realize the necessity of employing dentists in the army, navy and government hospitals. There can be no doubt, Congress, will upon due reflection, consider the dental surgeon as essential to the comfort and efficiency of the soldier as the army surgeon, at least in times of peace. Now will our task be done, should we get from Congress all we ask. We must labor to elevate our profession from the low plane on which it now moves to professional equality with the medical profession.

We must convince the learned fraternity, that the scientific skill requisite to preserve the dental organism in a healthy condition, is not less important than that required to cure a fever or saw off a leg, and then by our proficiency we must show them, that we keep equal pace in the proper exercise of that skill. And right here I would answer the question asked in the commencement of these remarks, viz: What is dentistry? It is the art of preserving the dental organs in a sound and healthy condition, and to a certain extent the health of the whole system, as all know how intimately the general health of the body is connected with that of the teeth. The best dentist, then, is he who understands the art of preserving the teeth. The multitude, however, call dentistry the art of stuffing and pulling the teeth, while the truth is, the extracting of teeth should not be reckoned as one of the legitimate duties of the dentist any more than of the doctor. Indeed, some of the most eminent dentists discard this branch altogether, and devote their skill to the more appropriate sphere of preserving the teeth. Dentistry, then, requires of the finished practitioner a knowledge of pathology and hygiene, the same as required of the physician, and for this reason if no other, the finished dentist should be regarded as the peer of the physician. But the perfect dentist must possess other qualifications. He must be a man of firmness and decision, of character pure, and like Cæsar's wife, above suspicion; of clean and temperate habits, for I need hardly picture to your minds how filthy, not to say dangerous, it is to inspect the teeth of a lady, as it is not unfrequently done by the thoughtless dentist, without washing his hand after it had been in the mouth of a tobacco chewer, or worse, in that of a syphilitic patient! The dentist should be temperate, because there is nothing so sickening to delicate ladies, or even to less sensitive men, as the breath of the habitual whiskey-drinker. In a word, the perfect dentist must be a gentleman and a scholar. To be properly educated, dental schools should no longer be separate from medical schools; they should constitute a department in all schools of medicine, so that the students of each department may have the benefits of the other. This course would effectually break down the wall of division, which now separates the dental and medical professions, and would place both on the same ground of equality. Let us then use our best endeavors to educate public sentiment and bring it up to this standard of thought. It will then soon be felt, that this system of education is an indispensable necessity, and will become of universal adoption. The great mass of the people is wofully deficient in the care and preservation of the teeth, and greatly need to be enlightened, for often our best efforts fail in keeping the dental organs in a healthy condition from lack of proper knowledge on this subject. But let not the young student be discouraged by the great work to be accomplished before he can become a perfect dentist, for he has only to bend all his talent and energies in the right direction to win the crown of ultimate success.

Besides the older and more experienced will gladly help the ambitious student, if he be of the right stamp, out of his difficulties, and give him proper instructions for his further guidance. Then be encouraged, young men, to press forward in the good work with untiring energy and zeal. And I would say to all, let no root of jealousy spring up among you, to mar the harmony of a united brotherhood, but let professional love and good fellowship be a bond of union between us, and let us all labor as one man, for the best interest of the whole. Then shall the Mississippi State Dental Association rank as high as Dental Associations North and East, and shall become an institution of honor and credit to the State and country.

